# Seasonal gaps in measles vaccination coverage in Madagascar

**DOI:** 10.1016/j.vaccine.2019.02.069

**Published:** 2019-04-24

**Authors:** K. Mensah, J.M. Heraud, S. Takahashi, A.K. Winter, C.J.E. Metcalf, A. Wesolowski

**Affiliations:** a Department of Ecology and Evolutionary Biology, Princeton University, Princeton, NJ, USA; b Virology Unit, Institut Pasteur de Madagascar, Antananarivo, Madagascar; c Woodrow Wilson School of Public Affairs, Princeton University, Princeton, NJ, USA; d Department of Epidemiology, Johns Hopkins Bloomberg School of Public Health, Baltimore, MD, USA

**Keywords:** Vaccination, Measles, Madagascar, Healthcare access

## Abstract

**Introduction:**

Measles elimination depends on the successful deployment of measles containing vaccine. Vaccination programs often depend on a combination of routine and non-routine services, including supplementary immunization activities (SIAs) and vaccination weeks (VWs), that both aim to vaccinate all eligible children regardless of vaccination history or natural infection. Madagascar has used a combination of these activities to improve measles coverage. However, ongoing massive measles outbreak suggests that the country was in a “honeymoon” period and that coverage achieved needs to be re-evaluated. Although healthcare access is expected to vary seasonally in low resources settings, little evidence exists to quantify temporal fluctuations in routine vaccination, and interactions with other immunization activities.

**Methods:**

We used three data sources: national administrative data on measles vaccine delivery from 2013 to 2016, digitized vaccination cards from 49 health centers in 6 health districts, and a survey of health workers. Data were analyzed using linear regressions, analysis of variance, and t-tests.

**Findings:**

From 2013 to 2016, the footprint of SIAs and VWs is apparent, with more doses distributed during the relevant timeframes. Routine vaccination decreases in subsequent months, suggesting that additional activities may be interfering with routine services. The majority of missed vaccination opportunities occur during the rainy season. Health facility organization and shortage of vaccine contributed to vaccination gaps. Children born in June were the least likely to be vaccinated on time.

**Discussion:**

Evidence that routine vaccination coverage varies over the year and is diminished by other activities suggests that maintaining routine vaccination during SIAs and VWs is a key direction for strengthening immunization programs, ensuring population immunity and avoiding future outbreaks.

**Funding:**

Wellcome Trust Fund, Burroughs Wellcome Fund, Gates Foundation, National Institutes of Health.

## Highlights

• First study exploring seasonality and added activities effects on routine vaccine.
• Routine immunization decreases in months surrounding vaccination campaigns.
• The majority of missed measles vaccine doses occurred during the rainy season.
• Specific birth cohorts are at risk to remain unvaccinated.
• Seasonal variation in health facility organization may shape vaccination gaps.

## 1 Introduction

Globally, considerable progress in reducing the burden of measles has occurred from the delivery of an effective, inexpensive, and fully immunizing vaccine [1]. However, since measles is highly transmissible, the World Health Organization (WHO) recommends that 95% of susceptible children be vaccinated to reduce transmission and prevent outbreaks [2]. Routine programs, designed to vaccinate children at least 9 months old at health facilities, are the backbone of the vaccine program and are designed to provide the most reliable coverage. Supplementary programs, often in the form of Supplementary Immunization Activities (SIAs), are coordinated efforts between national and international organizations that deliver mass vaccinations for all children within a designated age bracket, regardless of disease or vaccination history. These additional programs are intended to prevent measles outbreaks when routine coverage is insufficient, or to target particular high-risk groups, ages, and geographic areas [3], [4], [5]. Additionally, since 2011, the WHO African region has implemented an annual Vaccination Week (VW) that serves as an additional catch-up campaign.

Understanding how routine and additional programs interact and contribute to the successful vaccination of children is of clear programmatic interest, as this knowledge will inform vaccination program effectiveness, and aid in identifying gaps in coverage. Additional measles campaigns may negatively impact routine programs by redirecting healthcare workers [6], [7], [8], [9]. However, this effect has not been systematically quantified and is difficult to measure using commonly available ‘administrative coverage’ data (i.e., doses/target population). Additionally, the age of vaccination must also be taken into account, as vaccination may come too late relative to the average age of measles infection, or too early to be fully immunizing as a result of interactions with maternal immunity [10], [11]. In many low-income settings, road infrastructure, a lack of healthcare workers, vaccine dose shortages and challenges to cold-chain functioning affect vaccine delivery, reducing coverage in rural areas [12], [13], [14], [15]. Seasonal variability in access to care may also shape coverage [14], [16], [17], with gaps at particular times of year.

In Madagascar, measles-containing vaccine was first introduced in the 1970s [18] as part of the routine program. Since 2007, coverage has increased through a combination of three activities: (1) routine immunization (RI), (2) bi-annual VWs, and (3) SIAs every three years. The vaccination schedule was designed as a single-dose scheme, since up until 2017, Madagascar did not meet the criterion for a two-dose schedule (noting that the most recent WHO guidelines recommend that all countries include 2 doses regardless of coverage achieved [2]). Recent analysis of serological data from a convenience sample suggested that measles immunity may be around 83%, far from the 95% WHO goal [1], [4], [19], and reported national coverage (85–95%) may be correspondingly inaccurate [20]. Although Madagascar reported few measles cases in the last decade [19], an ongoing outbreak that started in September 2018, and that has thus far reached over 91 districts with more than 66,000 notified cases and more than 900 notified deaths, as of January 2019, confirms these results [21]. This crisis illustrates the need to understand how, when, and where coverage gaps may be occurring.

We analyzed nationally reported vaccination data and detailed individual level vaccination records to characterize vaccination coverage and missed opportunities across the country. We identify interactions between vaccination activities, and periods of disruption of RI. We conclude by discussing key factors that limit the effectiveness of vaccination programs in Madagascar and similar settings.

## 2 Materials and methods

### 2.1 Vaccination opportunities in Madagascar

**Fig. 1:**
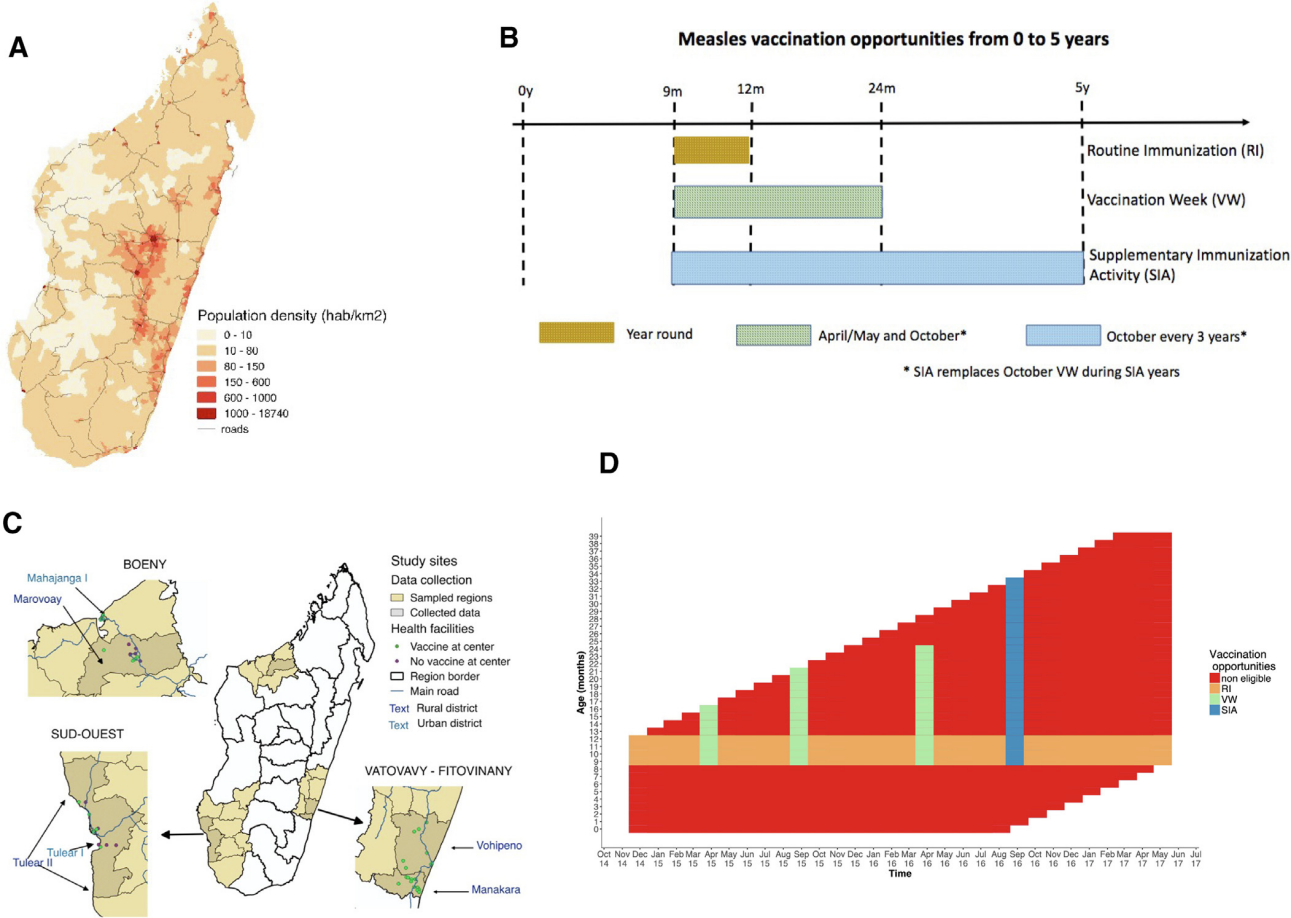
The study site and measles vaccination schedule in Madagascar. (A) Madagascar’s population density is highly variable, with few densely populated areas. (B) There are three measles vaccination mechanisms in Madagascar: routine immunization (RI), biannual vaccination weeks (VW), and Supplementary Immunization Activities (SIA) every three years. The eligible age range also varies by each program (RI: 9–12 months, VW: 9–24 months, and SIA: 0–59 months). (C) Vaccination cards and healthcare worker surveys were undertaken at 49 health centers from 6 districts throughout 3 regions across the country (Materials and Methods), yielding 15,951 cards digitized and 49 healthcare workers surveyed. (D) Differences in timing and age eligibility for vaccination leave children eligible for different vaccination activities throughout the year; and this characterization is used to extrapolate how vaccination coverage and its timeliness vary.

Madagascar has 22 regions and 114 districts with an estimated population of 26 million (Fig. 1A) [22]. The public health system includes three levels: a primary level with basic healthcare facilities, a secondary level with district and regional hospitals and a tertiary level with referral and university hospitals [23]. We focused on public sector data, noting that little data is available to characterize the role played by the private sector in providing vaccinations [24]. The number of facilities per district varies, with an average of 13.5 facilities per 100,000 people (Appendix Fig. S1A, S1B) and correlates with population density (adjusted R^2^ = 0.675, p < 0.01) (Appendix, Fig. S1C). RI is provided at the primary level with the exception of the BCG vaccine, which can also be given at birth to infants who were born at hospital and private clinics (secondary and tertiary levels).

Individuals aged 9–12 months are eligible for year-round RI delivered at primary health facilities (Fig. 1B). VWs are national campaigns designed to catch-up all children aged 9–24 months who have missed any vaccine including measles. These bi-annual campaigns (also referred to as ‘mother and child health weeks’) are performed predominantly at primary health facilities, but also can occur outside of the health facilities at households. SIAs occur every three years and recent campaigns have targeted children aged under 59 months. During SIAs, all children regardless of previous disease or measles vaccination history are vaccinated at primary health facilities and through outreach activities (Fig. 1B). We assigned doses delivered to different vaccination opportunities (RI, VWs, SIAs) based on the date of vaccination (Appendix, Tables S1, S2).

### 2.2 National administrative data on vaccine delivery

Data was provided by the Ministry of Public Health describing the number of doses of measles vaccine delivered per district per month from 2013 to 2016 (Appendix, Fig. S2A). The number of doses delivered was classified according to the age of the child (9–12 months, >12 months). Based on the date of the different opportunities, each month was classified as either RI, SIA, or VW depending on the type of vaccination opportunity scheduled (Appendix, Table S1), noting that VW and SIA doses may be over-estimated, since these activities typically last less than a month. To capture the effect of SIAs on dose delivery, we compared months following an SIA to the months before an SIA. Vaccination coverage estimates were calculated based on the number of doses given over the estimated target population. Administrative annual target populations (Appendix, Fig. S2B) were calculated from projected data estimated using a 3% linear growth rate since the most recent (1993) census and adjusted each year by an expert panel of health administrators based on previous doses delivered and deaths.

The rainy season per region was the month with the highest precipitation levels recorded by the National Direction of Meteorology of Madagascar (Appendix). We explored the degree to which the number of measles doses delivered *Y*, was explained by season, the presence of additional opportunities (VW or SIA), and region, using a multivariate linear regression (Appendix, Fig. S3):

$$log\left(Y_{ij}\right)=\beta_{1}x_{1ij}+\beta_{2}x_{2i}+\beta_{0j}+e_{ij,}$$

where x_1ij_ is an indicator for month *i* in region *j* being rainy; β_1_ captures the effect of a rainy month on doses delivered; this effect is assumed to be constant across regions. Here, x_2i_ is an indicator for month *i* containing an additional vaccination activity (activities are conducted at the national level so there is no regional variability); β_2_ captures the effect of a VW or SIA month on doses delivered. β_0j_ is a random effect for each region *j* and e_ij_ are the residuals.

To characterize variation in the number of doses delivered through RI, SIAs or VWs, these were compared via Chi-square and t-tests, with a significance threshold of 0.05. Correlations between population size and number of doses at regional and district level was characterized using a Pearson-correlation test.

### 2.3 Individual vaccination cards data

The majority of health facilities across the country store a vaccination card for each child that lists the date each vaccine dose was administered and birth date (see Appendix, Table S2). We analyzed vaccination cards from three regions representing low, intermediate and high population numbers (Appendix, Table S3). We collected 17,418 vaccination cards from 49 health facilities in six health districts (Fig. 1C). Although these data include children born between 2001 and 2017, most cards (16,031) are from children born from 2014 to 2016 since these children are still completing their vaccine schedule, and thus cards were consistently kept at the health facility. If the date of birth was missing, we assumed the child was born in the same month as their BCG vaccine (801 cards, 5% of all cards). Only 80 cards (0.5%) were excluded from the study due to missing dates (Appendix, Fig. S4A).

Children were considered eligible for routine measles vaccination between 9 and 12 months, and were classified into ‘timeliness’ groups accordingly, including ‘early’ (vaccinated before 9 months), ‘on-time’ (9–12 months), ‘late’ (after 12 months), or ‘unvaccinated’ (i.e., eligible but unvaccinated at the time of data collection) (Appendix, Fig. S4A, S4B). The vaccination mechanism is not recorded; however, we assumed that if the date of vaccination corresponded to a VW or SIA, the child was vaccinated through this mechanism instead of RI. It is likely that vaccination performed outside healthcare facilities was under-recorded on vaccination cards, reflecting real-life conditions on vaccination data accuracy. Our estimates of coverage are thus likely to be conservative. Vaccination cards do not record multiple doses of measles vaccine, so we were not able to evaluate second doses.

We used the date of birth to create monthly birth cohorts (Appendix, Fig. S4C) from January 2015 to March 2017. For each timeliness group, we calculated the proportion of eligible children who were vaccinated per month and the average proportion of children who were vaccinated through routine vaccination (see Appendix, Tables S4, S5). Since the direct effects of additional activities were difficult to measure (denominator data is lacking and numerator data is likely under-reported) we focused on temporal variation in routine immunization. We identified 3 time periods: post-May VW (May to September 2015), post-October VW and SIA (October 2015 to April 2016), and post-May VW (May to September 2016) and used an ANOVA test and multivariate linear regression to identify the impact of additional activities on RI (Appendix Table S6, S7, Fig. S5).

Following standard comparisons e.g., of age and proportions within timeliness groups across geographical settings and months across the year, opportunities for vaccination were assessed with a Lexis diagram [25], [26] with cohorts created from birth months, events (RI, SIA or VWs) from national data, and vaccination status from vaccination cards (Fig. 1D).

### 2.4 Healthcare worker survey

We surveyed healthcare workers at the facilities where vaccination cards were digitized (Appendix, Tables S8, S9) about (1) geographic access to healthcare, (2) vaccine supply chain, (3) the organization of the routine program, and (4) staffing for both routine and additional activities (see Appendix, Table S8 for complete survey). Associations between facilities’ characteristics and number of children who were vaccinated early, late or remained unvaccinated was performed using Lasso regression. Each facility characteristic was ranked 1 to 4, with 1 reflecting the least desirable situation and 4 the most desirable. We then assigned a weight to each facility characteristic according to their Lasso coefficient (Appendix, Table S10). We then created three scores for each health facility, one for each vaccination scenario (late, early, or unvaccinated), that we used to visualize health facilities’ barriers to timely vaccination. Statistical analyses were done with R version 3.4.3; maps were produced using QGIS 2.18.

## 3 Results

### 3.1 National administrative data on dose delivery

**Fig. 2:**
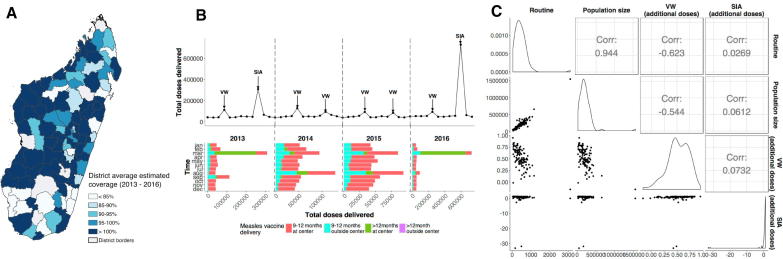
Measles vaccination dose data. (A) District level administrative vaccination coverage (number of doses divided by the estimated target population size), 2013–2016; average coverage per district has increased from 72% in 2013 to 200% 2016; values over 100% indicate target population size uncertainty, or issues of revaccination of the same children. (B) Number of doses delivered per month by timeliness group, indicating increases in numbers of doses delivered during additional activities (peaking during the SIA) and declines during the rainy season (lowest recorded levels, Appendix). The majority of doses are given as part of routine vaccination on time (aged 9–12 months). Months that included an SIA were when the largest number of doses were delivered outside of the health facility (ANOVA, F(2, 12) = 11.88, r^2^ = 0.62, p = 0.001) and when children were most likely to receive a late (over 12 months of age) vaccination. Children who received their vaccination late (over 12 months) were predominately vaccinated during an SIA month. (C) Correlation matrix showing the relation between doses delivered across districts from 2013 to 2016 through routine immunization, population size, additional doses delivered through VW and SIA.

From reported national data (Fig. 2A, B, Appendix, Table S11), an estimated 3.7 million doses were delivered to a target population of 3.2 million (Materials and Methods), suggesting that every child aged 9–12 months was vaccinated (118%). At the regional level yearly ‘administrative coverage’ (i.e. doses/target population) ranges from 66 to 275% (Table 1), and in all but 11 districts, coverage regularly exceeds 100% (Fig. 2A), suggesting that administrative estimates of target population size are inaccurate (Materials and Methods). To mitigate these biases, we focus on the number of doses delivered.

**Table 1:** Number of doses delivered by region from 2013 to 2016. Doses are shown target population, estimated coverage and relative difference between average doses in routine and during additional activities.

Region	Population size	Total number of doses	Total target population	Coverage (%)	Relative difference RI/VW (%)	Relative difference RI/SIA (%)
Alaotra Mangoro	1,118,326	207,020	154,954	134	64	1255
Amoron'i Mania	781,715	111,749	109,964	102	87	718
Analamanga	3,778,682	474,449	438,082	108	19	598
Analanjirofo	1,109,983	174,360	162,451	107	104	661
Androy	788,786	61,707	93,241	66	305	890
Anosy	736,252	101,309	96,778	105	113	655
Atsinanana	1,395,039	201,297	185,902	108	60	685
Betsiboka	314,888	55,972	44,009	127	167	1167
Boeny	878,193	109,889	103,233	106	131	627
Bongolava	497,432	116,041	69,617	167	104	2775
Diana	767,684	98,082	100,137	98	162	698
Haute Matsiatra	1,311,621	184,674	172,631	107	78	595
Ihorombe	334,189	119,671	43,557	275	171	5856
Itasy	789,054	148,505	106,182	140	56	1494
Melaky	313,855	78,413	41,645	188	362	3776
Menabe	650,942	185,774	92,858	200	248	4161
Sava	1,062,976	145,745	151,919	96	68	134
Sofia	1,342,241	202,795	176,676	115	271	944
Sud-Est	964,824	109,575	124,381	88	235	707
Sud-Ouest	1,451,997	189,167	183,684	103	132	601
Vakinankaratra	1,940,894	329,825	271,582	121	64	776
Vatovavy Fitovinany	1,523,416	228,798	218,201	105	126	429

The majority of doses delivered per year were given as part of RI (83%), followed by the VW months (8–17%), and SIA months (8%), although this did vary temporally (Fig. 2B). The smallest number of RI doses was delivered from November to January which corresponds to the period of heavy rains (Fig. 2B, Multiple linear regression, F(23, 5192) = 112, p < 0.001, R^2^ = 0.30, Table S12). The majority of children were vaccinated on time (9–12 months, 76%). Of children vaccinated late, most were vaccinated during an SIA month (86.9%), indicating that the catch-up goal of additional vaccination opportunities was being achieved (ANOVA, F(2,12) = 7.409, p = 0.008, r^2^ = 0.478).

We compared doses delivered during months following an SIA to doses delivered during months before an SIA (Methods, Appendix, Table S13). The monthly average number of doses delivered through routine immunization (M = 1025.3, SD = 966.2) was significantly lower in months following an SIA (M = 727.2, SD = 678.1, t(1932.1), p < 0.001), suggesting a potential disruptive effect of SIAs (Appendix, Table S14). Surprisingly, the average number of doses delivered in the months surrounding a VW were not different before and after VWs (Student test, test, t(1693.6) = 0.192, p = 0.847), suggesting, either that VWs do not cause disruption or that the aggregated data does not include sufficient temporal detail to disentangle impacts on routine immunization from VW doses.

We explored spatial differences in the relationship between RI, SIAs and VWs (Material and Methods). The average number of doses delivered during VWs was negatively correlated with the average number of doses delivered during RI suggesting that where RI was performing well, fewer children may require catch-up doses (Fig. 2C). Additionally, more populated locations had the lowest relative VW to RI doses (Fig. 2C).

### 3.2 Individual-level vaccination history data

**Fig. 3:**
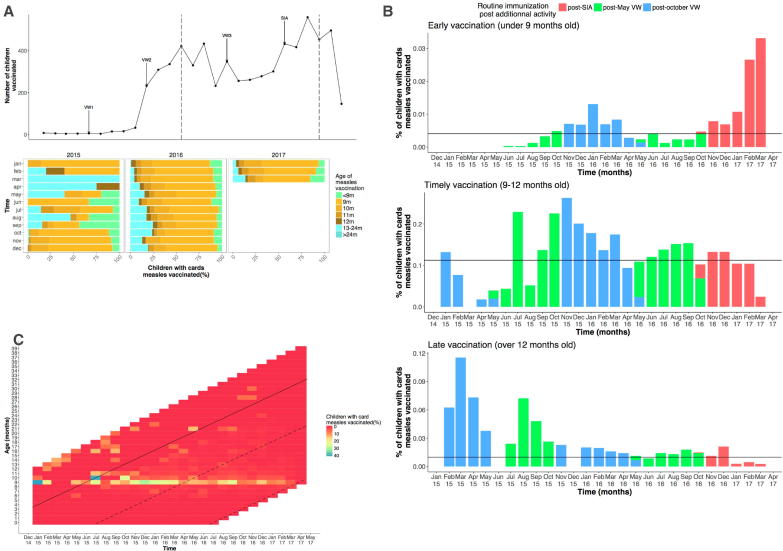
Vaccination coverage estimates based on digitized vaccination cards from 49 health facilities showing (A) total number of children vaccinated per month by age (top panel) with low numbers in 2015 attributable to limited data, although increases in numbers vaccinated during either a VW or SIA are still detectable. Most children are vaccinated on time (lower panel); occurrence of early or late vaccination varies throughout the year (colors, legend). (B) Proportion of children vaccinated through routine immunization on time, early, or late in each month, showing significantly low timely vaccination in March and April. (C) Percentage of children with cards who are vaccinated by age and birth month, with birth cohorts shown from ages 0–39 months. Children born in June (dotted line) are less likely to be vaccinated than other birth-month cohorts (less red). Children born at the end of the year had the highest overall vaccination levels.

Children with vaccination cards at sampled health centers (Appendix, Fig. S6) who were vaccinated mostly received timely vaccination (81%) and were vaccinated through RI (95%) (Table 2). As in the national data, higher than expected numbers of doses are delivered during months that include either an SIA or VW (Fig. 3A), but the difference was less pronounced than in the national dose data. In contrast to national data, doses given during the exact week of a VW activity were identifiable in the individual data (see Methods).

**Table 2:** Repartition of children with cards by timeliness group and vaccination opportunities.

	Children with cards (N = 11,976)^a^	%
Vaccinated children	6335	53
Timely vaccination	5125	42.8
Early vaccination	540	4.5
Late vaccination	670	5.6
Unvaccinated (+9 months)	5641	47

*Vaccination opportunities*		
Routine immunization	6017	95
VW	138	2.2
SIA	176	2.8

a Here are presented data for children over 9  months by the end of our study.

The highest rates of early vaccination occur after the October 2016 SIA (Welch-corrected ANOVA, F(2,16.8) = 3.57, p = 0.004, R^2^ = 0.33, Appendix, Table S15), from November 2016 to February 2017 (Fig. 3B). Additional activities (such as the May or October VW/SIA) may fulfill the aim of children receiving a second dose, however it was not recorded on the vaccination cards. Timely routine vaccination has the largest relative reduction, before an SIA, in March (Student-test, t(36) = −5.33, p < 0.001) and April (Student-test, t(24) = −2.11, p = 0.04, Fig. 3B) suggesting that the May VW may be interrupting routine activities (Fig. 3C). Finally, of the 47% of children who remained unvaccinated, but eligible at the end of our study, the most common birth month was June (16%). Children in this birth cohort face a number of barriers to vaccination including entering routine eligibility age in March/April (months with low routine coverage) (Fig. 3C).

However, the ability of these campaigns to catch up older children varies spatially (Appendix, Fig. S7). In rural areas, the average age of vaccination is higher (10.1 months vs 9.8 months, student-test t(5083.5) = 3.862, p < 0.01), as is the proportion of unvaccinated children (52.1% vs 34.6%, χ^2^ = 372.5, p < 0.001) suggesting that these locations are more reliant on catch-up campaigns to achieve vaccination of children. Overall, vaccination opportunities in children over 12 months are low throughout the country, particularly during months offering only routine services.

### 3.3 Healthcare factors related to missed vaccinations

**Fig. 4:**
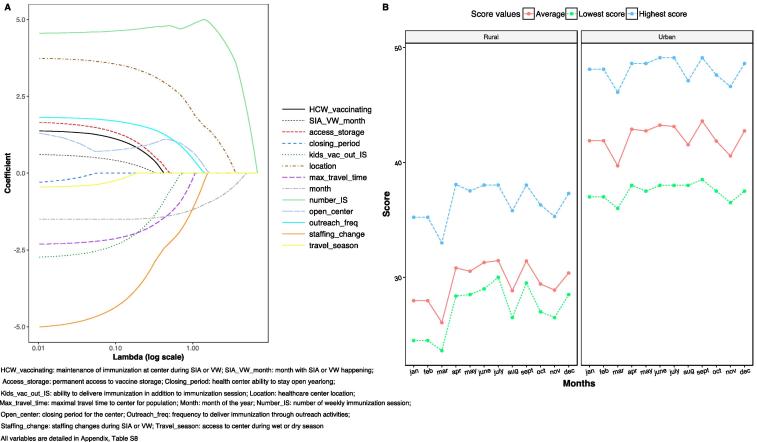
Health facility factors related to a high number of unvaccinated children. (A) A lasso regression comparing the penalty coefficient (x axis) by beta coefficient (y axis) indicates that the five most important variables to explain the number of unvaccinated children were: the number of additional, routine outreach activities, the facilities location, the ability of the facility stay open year-round, and the frequency of outreach activities. (B) Each facility ranked by vaccination access score through time (per month) based on the regression (see Materials and Methods) with red indicating poor access and blue indicating good access. Facilities are classified by location from urban areas (top rows), those on a main road but rural (middle rows), and those on a secondary road and rural (bottom rows). Across all facilities, the lowest vaccine access scores were observed in March, August, October and November suggesting that seasonality in access may be impacting areas of the countries with diverse climatic patterns. Interestingly, some health facilities in rural areas had a similar score to those in urban areas suggesting that the simplistic urban-rural divide for access is more nuanced.

To identify which factors at health facilities, led to seasonal coverage gaps, we conducted a survey in health centers where the vaccination cards were digitized (Fig. 1C, see Appendix, Table S8). A Lasso regression (Fig. 4A) indicated that the most significant factor determining the number of unvaccinated children was the frequency of immunization sessions: daily, biweekly, weekly, monthly, or unknown. Other significant factors were: geographic location of the health facility (urban, rural on main road, rural on secondary road), the ability of the health facility to remain open during additional activities, the frequency of health facility closure, and vaccine storage capacities. Centers that were unable to stay fully staffed year-round, and unable to have permanent access to vaccine were those most likely to have a high number of unvaccinated children with qualitatively similar patterns identified for early and late vaccination (see Appendix, Fig. S8).

We used these results to construct a monthly varying score integrating health facility characteristics that determine the number of unvaccinated children (Material and Methods). This score decreased during March, August, October and November, which aligns with additional vaccination activities. The average health facility performance score was higher in urban areas (M = 33.118 SD = 3.017) than in rural areas (M = 26.539, SD = 3.775, t(4 9 8) = −23.012, p < 0.001). However, considering the extensive heterogeneity in both urban and rural facilities performance scores and the fact that many facilities in rural areas had comparable performance to facilities in urban areas (Fig. 4B), we conclude there is no clear difference between rural and urban health facilities’ performance (Fig. 4B).

## 4 Discussion

Understanding the interaction between routine and additional vaccination activities will contribute to successfully eliminating measles [3], [27]. In Madagascar, national data indicates that additional activities (SIAs and VWs) contribute a substantial number of doses delivered, but also modulate RI during months following these activities [6], [7], [8], [9]. This relationship could reflect successful activities, especially SIAs, leaving fewer children unvaccinated, and hence driving a reduction in the need for routine coverage. However, it could also be the result of health facilities’ inability to conduct both an SIA and routine vaccination, with many resources diverted to the SIA campaign as was reported in Cameroon [7]. Additionally, this effect may be partially confounded with seasonal access to healthcare, since the lowest number of doses delivered corresponded to the rainy season in Madagascar.

The national administrative data do not identify the exact route of vaccination and exact age of vaccination. Using individual vaccination cards that recorded the date of the first dose of measles containing vaccine revealed that a high proportion of eligible children (47%) remained unvaccinated, suggesting a high risk for a measles outbreak in Madagascar. Timely routine vaccination (9–12 months) decreased after SIAs, suggesting a negative interaction between routine and additional activities. The large proportion of children who were vaccinated early and would need a 2nd dose after 9–12 months, given interference with maternal immunity, supports that updating the vaccination card to record second doses would help to fill the age gap in routine immunization. It would also provide incentive for healthcare workers to catch-up children while aligning with WHO measles vaccination recommendations. In our data, late vaccination primarily occurred during the additional activities (SIAs and VWs) and decreased after the October VW, implying that children missing these activities may have few chances to be vaccinated. We also identified a specific birth cohort (children born in June) who are the most likely to have missed various vaccination opportunities. Overall, Madagascar seems highly reliant on additional activities to catch-up children who may not have received their routine vaccination on time, or at all.

The recent measles outbreak suggests that Madagascar was experiencing a honeymoon period [28] and the low immunity levels from inadequate vaccination coverage resulted in a large proportion of the population susceptible to measles infection [29]. Our results may help to prevent future outbreak as they show when and where routine immunization could be reinforced to prevent missing opportunities to increase population-level immunity. A description of healthcare facility characteristics revealed that more remote facilities had a higher percentage of children who were not vaccinated on time, reflecting the well-known barrier of travel time to healthcare access [7], [30], [31]. Analyses indicated that health facilities that could maintain routine immunization during a supplementary program, had a permanent vaccine supply, and had more staff members had the fewest unvaccinated children highlighting the importance of healthcare functioning during the rainy season. These may be important avenues to improve overall vaccination rates and suggest that small changes such as providing permanent vaccine access via a solar fridge and increasing the number of healthcare workers during additional campaigns to maintain staffing at health facilities may improve vaccination coverage.

Limitations in the granularity and detail in both vaccination data sets are a caveat of our analysis. We were unable to account for additional vaccine doses (beyond the first), since these were not recorded. This may be partially responsible for the mismatch between estimates in doses given during an SIA or VW estimated from the national data versus the vaccination cards. Vaccine doses given as part of an SIA or VW were identified based on the date, limiting our ability to directly measure their impact on routine services. Monthly aggregated national data did not allow us to disentangle routine immunization from additional activities when both occurred in the same month. As a result, we may have underestimated the effect of VWs on routine vaccination, explaining the discrepancy with the vaccination cards’ data. Moreover, individual data provided more precision on vaccination prior to the SIA period, another potential source of the discrepancy in inferred effects. Finally, we digitized vaccination cards that were available at health facilities, but record-keeping likely varies between facilities, preventing facility to facility comparison. Further, only short-term temporal trends could be assessed, since only cards for children born within the last few years are kept at facilities. Relying on vaccination cards may have led to an underestimation of the proportion of children vaccinated, as all children in the center catchment area might not have been included in our study, or vaccination delivered outside health facilities may not have been reported on cards. Finally, although our analysis encompassed a large array of settings, our individual data may be biased because considerable spatial heterogeneity in Madagascar means that there may be additional seasonal patterns and issues of vaccination programs that our study did not include. In particular, the southern and northern areas of the country were not explored in detail, and may feature region-specific factors related to healthcare access and immunization. However, consistency of the findings across the three very different regions suggests that our findings could be generalizable across the country.

Our detailed investigation of the routine vaccination program in Madagascar characterizes spatial and temporal differences in how children are vaccinated, and timeliness of vaccination, with implications for introduction of additional vaccines such as rotavirus. Evaluating how the interacting seasonal components of immunization have effects on the variation in age eligibility for vaccination (which will vary across antigens), will be an important line of information in evaluating how to prevent pockets of unvaccinated children. Further, evidence for interactions between additional activities and RI for measles suggest a need for evaluating this phenomena for other antigens delivered via multiple campaigns, whether scheduled (such as VWs or SIAs), or outbreak response (such as poliovirus outbreaks [32], [33]).

## Contributors

Conceived and designed the experiments: K.M., C.J.E.M. and A. Wesolowski. Performed the analysis: K.M. Contributed reagents/materials/analysis tools: K.M., J.M.H A. Winter, S.T., C.J.E.M and A. Wesolowski. Wrote the paper: K.M., J.M.H., A. Winter, S.T, C.J.E.M and A. Wesolowski.

## Declaration of interest

The authors have no conflicts of interest to report.
