# Supplementary material for: Seasonal gaps in measles vaccination coverage in Madagascar
Source: Vaccine. 2019 Apr 24;37(18):2511–9. doi: 10.1016/j.vaccine.2019.02.069 (PMC6466641; doi:10.1016/j.vaccine.2019.02.069)
Supplement: Supplementary data 2 [file mmc2.pdf]

Seasonal gaps in measles vaccination coverage in Madagascar

Supplementary Information

K. Mensah[1], J.M. Heraud[2], S. Takahashi[1], A.K. Winter[1], C.J.E. Metcalf[1,3*], A. Wesolowski[4*]
[1] Department of Ecology and Evolutionary Biology, Princeton University, Princeton, NJ, USA.
[2] Virology Unit, Institut Pasteur de Madagascar, Antananarivo, Madagascar.
[3] Woodrow Wilson School of Public Affairs, Princeton University, Princeton, NJ, USA.
[4] Department of Epidemiology, Johns Hopkins Bloomberg School of Public Health, Baltimore, MD, USA.

1. Methods

1. Health facilities in Madagascar

The geographic location of primary public health facilities was obtained from the Institut Pasteur of Madagascar. Figure S1A shows the density of health facilities by regions.
Using estimated population data from WorldPop (www.worldpop.org), we calculated the density of health facilities per 100,000 individuals (see Fig S1B). There is a strong correlation between the number of health facilities and population density per region (see Fig S1C, Pearson's correlation, adjusted R^2= 0.68, p<0.01).

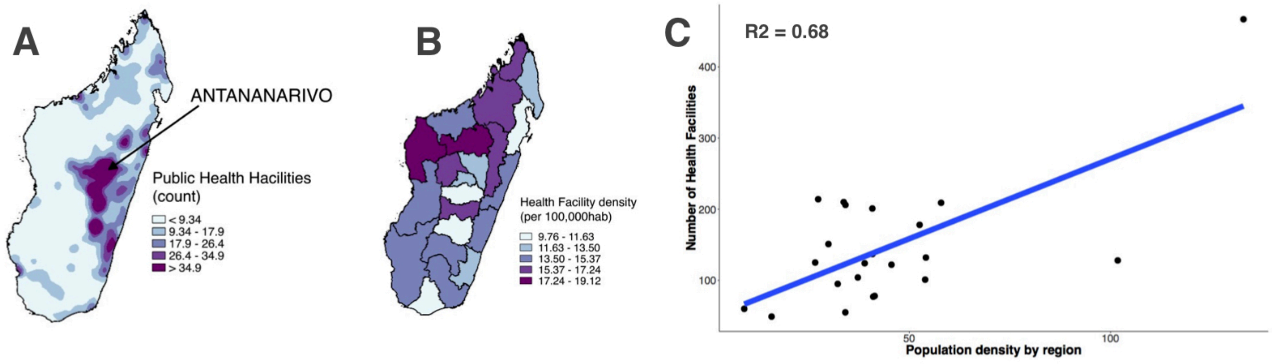

Figure S1: The public primary healthcare facilities in Madagascar. A) The total number of public health facilities per region is shown. **B)** The density of health facilities per 100,000 individuals and due to variable population density, some regions have a high density, although small absolute number of facilities. **C)** Population density is strongly correlated with the total number of health facilities in a region.

2. Vaccination activities in Madagascar

From 2013 to 2016, Madagascar performed two SIAs (Supplementary Immunization Activities) and six VW (Vaccination Weeks) as shown in Table 1. Doses delivered during the corresponding month were assigned to either an SIA or VW. All other doses were assumed to be delivered through routine immunization. All primary health facilities (see Fig S1) perform vaccinations at the health center. In an area where the facility's catchment area is greater than 5 km, health care workers also perform outreach activities as part of their routine activities. In this instance, they will vaccination children directly at their households instead of the health facility.

Table S1: The additional vaccination activities in Madagascar from 2013 to 2016. The date, type of activity, and age target for non-routine activities is shown.

Date	Activity	Age target (months)
**22/04/2013 - 26/04/2013**	VW	9-24 months
**13/10/2013 - 18/10/2013**	SIA	9-59 months

**05/05/2014- 09/05/2014**	VW	9-24 months
**27/10/2014 - 31/10/2014**	VW	9-24 months
**11/05/2015 - 15/05/2015**	VW	9-24 months
**28/10/2015 - 08/11/2015**	VW	9-24 months
**09/05/2016 - 13/05/2016**	VW	9-24 months
**26/10/2016 - 30/10/2016**	SIA	9-59 months

Table S2: The national vaccination schedule in Madagascar.

Vaccination age	Vaccine	Number of doses
**At birth**	BCG	1
	Polio	1st
**At 6 weeks**	Polio	2nd
	Diphteria/Tetanus/Pertussis/Hepatitis B/Hemophilius Influenza B	1st
	Pneumoccocal Vaccine (PCV-10)	1st
	Rotavirus	1st
	Vaccine Polio Inactivated	1
**At 10 weeks**	Polio	3rd
	Diphteria/Tetanus/Pertussis/Hepatitis B/Hemophilius Influenza B	2nd
	Pneumoccocal Vaccine (PCV-10)	2nd
	Rotavirus	2nd
**At 14 weeks**	Polio	4th
	Diphteria/Tetanus/Pertussis/Hepatitis B/Hemophilius Influenza B	3rd
	Pneumoccocal Vaccine (PCV-10)	3rd
**At 9 months**	Measles	1

3. National vaccine dose data for measles vaccine in Madagascar

The estimated target population is likely inaccurate in Madagascar. The most recent census was conducted in 1993 and all subsequent population estimates are projections using a 3% annual growth rate. The target population is further adjusted by an expert panel of health administrators' in country based on the previous years' number of doses delivered and deaths. The variation in target population estimation by region is shown in Fig S2B.

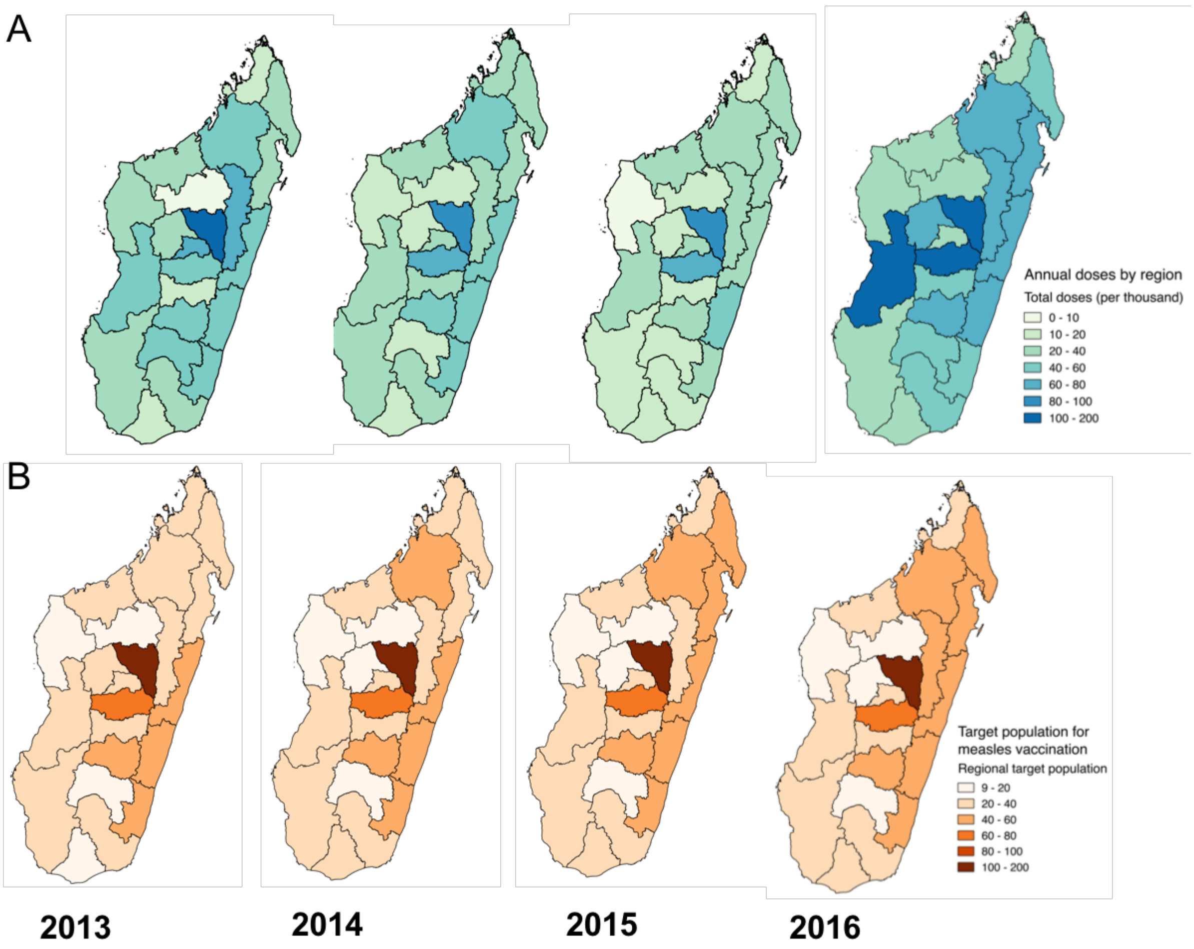

Figure S2: The geographic distribution of the number of measles doses delivered from 2013-2015 and the estimated target population. A) The annual number of doses delivered per year. **B)** The estimated annual target population has remained fairly consistent between years.

4. Identifying rainy and dry seasons in Madagascar

The National Direction of Meteorology of Madagascar publishes biannual precipitation data per region and calculate for each month the regional mean from 1981 to 2010. Using these data, we assigned a dummy variable (rainy or dry season) to each month by region if precipitations were over the mean : http://www.meteomadagascar.mg/tendance-saisonniere
To cross-validate this data, we observed the level of precipitation in Madagascar using the African flood and drought monitor tool: http://stream.princeton.edu/AWCM/WEBPAGE/interface.php?locale=en
The rainy season was defined if more than 70mm was recorded based on the Princeton Rank Scale (1).

i. Exploring the association between rainy season and number of doses delivered by regions

We explored the seasonal effect of precipitation on number of doses delivered by region using multivariate linear regression. We used a rainy season dummy variable (per month), region (categorical with 22 levels), and a dummy variable for an additional activity if it occurred during the month (VW or SIA). The distribution of the untransformed variable (number of doses delivered) was right skewed and we log transformed the data to reduce the skewness. Overall, the regression model was able to explain 30% of the variance in the number of doses delivered (see Fig S3).

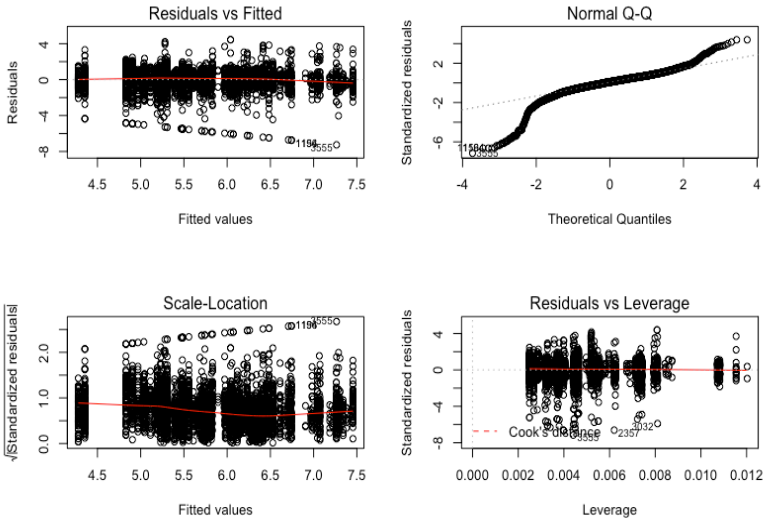

Figure S3 – The diagnostic plot of multivariate linear regression exploring the association between the monthly number of doses delivered and the rainy season. Log transformed variable.

5. Health facility vaccination coverage data

In order to understand vaccination coverage in various areas of Madagascar, we analyzed individual vaccination card data from three regions in different areas of the country. To be as much representative as possible, we choose three regions with three different population density level and administrative coverage level (Table S3).

Table S3: Population size and administrative measles coverage in study sites regions

Region	**Population density (hab/km^2)**	**Administrative measles vaccine coverage in 2015**
**Boeny**	26·61	88%
**Sud-Ouest**	10·78	76%
**Vatovavy**	57·89	82%

In each region, we identified one urban (that contained a city) and one rural district. Using the list of healthcare centers located in each district, we used block randomization to identify 8-10 centers based on the WHO routine immunization assessment guidelines (2). Due to security concerns or the current road condition, some centers were deemed inaccessible. In total, we visited 50 health centers, although one facility did not store vaccination cards and was excluded from the analysis.

In each health facility, paper vaccination cards are stored and were analyzed for children born between 2014-2015. Data were digitized and anonymized to remove any identifiable information. In the majority of cases, cards were kept until the end of the current year or until children receive their measles vaccination. We calculated the age of vaccination based on the child's birth date. If the child's birth date was missing, we assumed that the date of the BCG vaccine was the birth date. A flow chart of our data is presented in Fig. S4A.

Our data and the Demographic Health Survey (2004 to 2009) both did not show any seasonality in births (Fig S4B), suggesting that our findings were not cofounded by the birth seasonality.

Children were considered as vaccinated against measles if the date of vaccination was listed. Children were classified in timely, early, late, un-eligible or unvaccinated based on their age at vaccination or if they were unvaccinated (see Fig 4A) and varied by health facility (see Fig S4C). In each month of data, we calculated the number of eligible children for vaccination by age group (see Fig S4D).

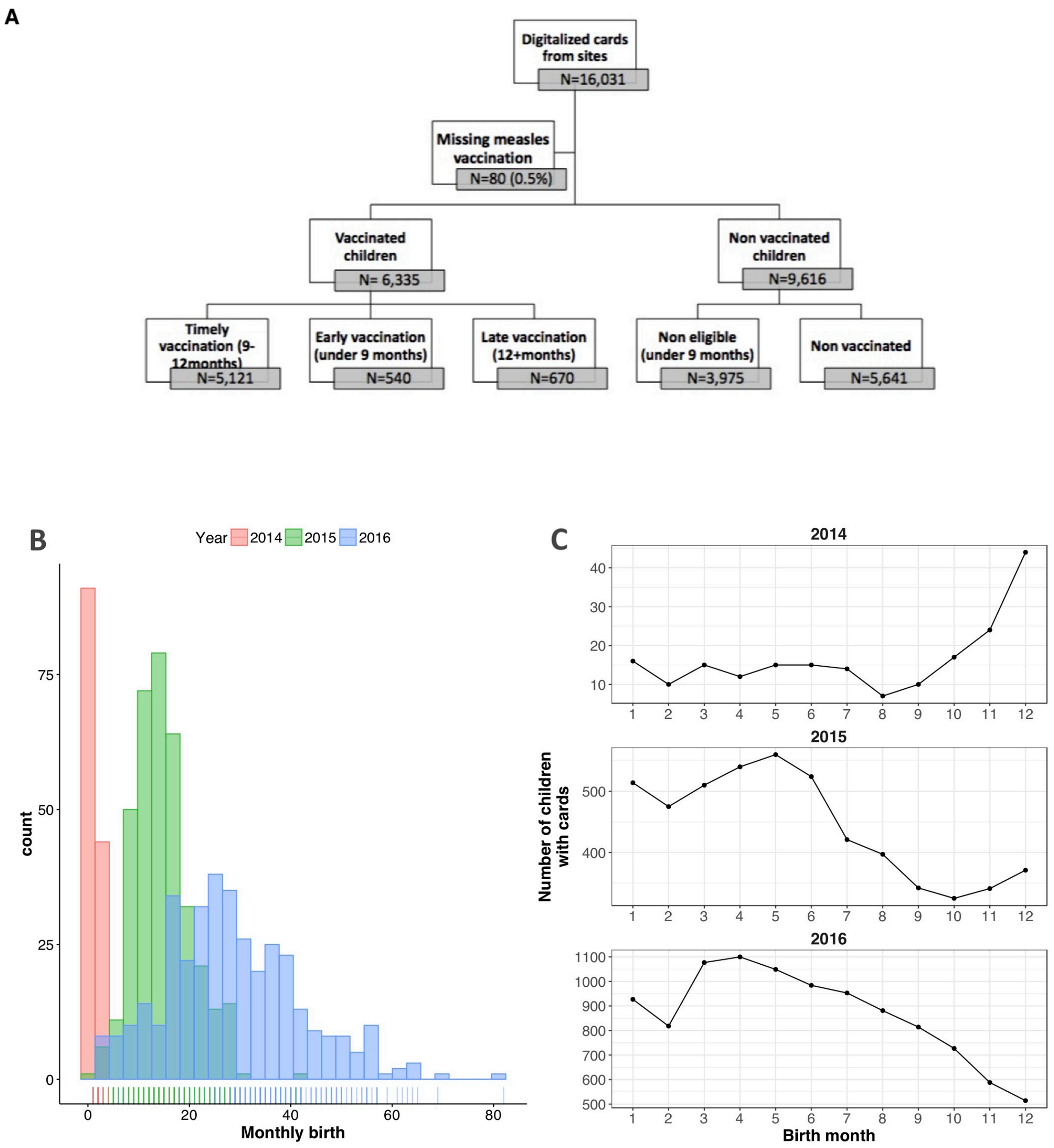

Figure S4: The individual vaccination card data. A) Individual vaccination cards were digitized from health facilities and classified as time, early, late, not eligible, and not vaccinated. **B)** The number of births per month based on the vaccination card data. C) Number of births per month per year based on vaccination cards.

Table S4: Number of children with cards and denominator use to calculate proportion of vaccinated

	Timely		Early		Late	
**Month**	Number of children with cards who were vaccinated for measles	Number of children with vaccination cards	Number of children with cards who were vaccinated measles	Number of children with vaccination cards	Number of children with cards who were vaccinated for measles	Number of children with vaccination cards
**JAN**	779	5416	82	5722	20	5464
**FEB**	710	5587	70	4924	45	6793
**MAR**	476	5421	58	4335	42	7554
**APR**	193	1980	16	4330	27	1576

**MAY**	274	1854	23	4894	56	2157
**JUNE**	205	1608	30	4918	24	2599
**JULY**	216	1432	10	5665	49	3198
**AUG**	216	1366	21	4938	56	3270
**SEPT**	225	1397	29	7737	79	4097
**OCT**	492	2328	66	8233	106	4479
**NOV**	604	3105	65	6875	57	4780
**DEC**	734	4272	51	6380	109	4968

Table S5: The number of children with cards and the denominator use to calculate average proportion of proportion of vaccinated for each timeliness group

Timeliness group	Number of children with cards who were vaccinated for measles	Number of children with cards	Average proportion vaccinated through routine (%)
**Early (under 9 months)**	493	121164	0.004
**Late (over 12 months)**	605	61366	0.009
**Timely (9-12 months)**	4903	43580	0.113

The variation in routine immunization following any additional activity (VW or SIA) were analyzed using one-way ANOVA and multivariate linear regression.
We identified 3 time-periods based on the time following any activity (Table S2, Table S4) and excluded the vaccination done during a VW or SIA.

Table S6: Time period used to analyze variation in proportion of children with cards measles vaccinated

Time period	Months
**Post May VW**	June 2015 – September 2015
	June 2016 – September 2016
**Post October VW**	November 2014-April 2015
	November 2015-April 2016
**Post SIA**	November 2016-April 2017

An ANOVA was performed to explore association between the coverage and the different time period. We checked if the assumptions of variance normality and variance homogeneity as true using Shapiro and Levene Tests (Table S7). Transformations were used to achieve normality (log, square root, and arcsine transformations). Through arcsine transformation, normality was reached for all three timeliness categories and variance homogeneity was reached only for early vaccination. To overcome heteroskedacity, we decided to perform a Welch-corrected ANOVA on arcsine-transformed data for timely vaccination (Fig S5A), early vaccination (Fig S5B) or late vaccination (Fig S5C).

Table S7: Shapiro Test and Levene Test checking normality and homogeneity of variance before performing ANOVA to explore relation between percentage of children with cards vaccinated through RI and the period post- additional activity

	Shapiro test	Levene Test	Shapiro test	Levene Test	Shapiro test	Levene Test
	Timely vaccination		Late vaccination		Early vaccination	

**% children with cards vaccinated by time period**						
**Untransformed**	0·371	0·182	0·001	0·252	0·014	0·007**
**Log transformation**	< 0·01	0·193	0·002	0·254	0·015	0·007**
**Square root transformation**	0·377	0·187	0·002	0·253	0·015	0·007**
**Arcsin transformation**	0·320	0·234	0·673	0·457	0·709	0·024*

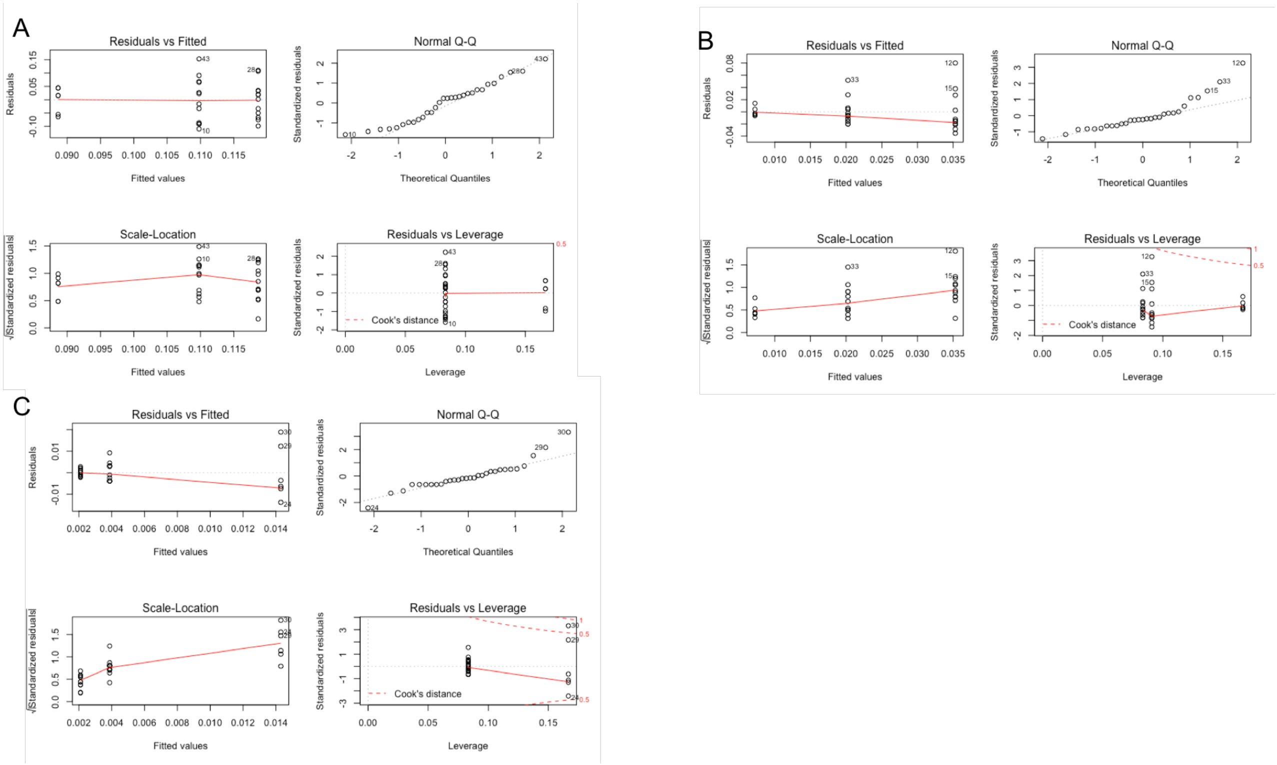

Fig S5: The diagnostic plot, ANOVA relation between percentage of children with cards who were vaccinated through RI and months following each activity Results are shown for the A) timely vaccination. B) late vaccination. C) early vaccination.

6. Healthcare facility data

Based to their location, health facilities were considered as urban, rural on the main road or rural on the secondary road (see Fig 1C and Table S9). The only exception was Manakara which is considered a rural district even though a major town is included. In this case, the health center in town was considered as urban while all the other were considered rural. To identify the relation between number of early, late or unvaccinated children and health centers' characteristics, we performed a Lasso regression through a Gaussian linear regression. LASSO regression was chosen because of its ability to shrink coefficients of variables that do not contribute information to the model to zero, eliminating the need to do features selection on high dimensional data (3). At each health facility, a health facility worker provided information about staffing, operating hours, and the frequency of vaccination activities (see Table S8). These data were used as covariates for each health facility. For each timeliness group (early, late, on-time and unvaccinated), we performed a conservative analysis with all the variable before removing the overlapping variable. We used the lasso results to rank our variables and weighted them assigning to each variable its coefficient (Table S10).

Table S8: Health worker survey

Variable	Question	Possible Responses	Label
	**Healthcare access**		
**Location**	Where is the center located?	urban area	3
		rural, main road	2
		rural, secondary road	1
**Open center**	Is the center open?	yes, all the time	3
		some closing periods	2
		No	1
**Closing period**	When do you have closing periods?	Never	4
		Quarterly review	3
		Healthcare workers (HCW) training	2
		Healthcare workers leave (holiday, sick, other)	1
**Max_travel_dry**	What is the maximal travel time for population?	<1h	3
		1-2h	2
		>2h	1
**Travel_season**	When is this travel time happening?	Dry season	2
		Rainfall season	1
	**Vaccine availability at center**		
**Access_storage**	How often do you have access to vaccine storage facility? (district level or other health center)	daily	3
		weekly	2
		monthly	1
**Track_vaccine**	Do you track vaccine cold chain?	Yes	3
		sometimes	2
		never	1
	**Routine immunization organization**		

**Number_is**	What is the number of weekly immunization session?	5/5	4
		2/5	3
		1/5	2
		<1/5	1
**Kids_vac_out_is**	Are kids getting vaccinated if they are coming other days than regular immunization session?	Yes	2
		No	1
**Outreach_freq**	What is the frequency of outside of center routine immunization?	>1month	4
		1/month	3
		<1/month	2
		Never	1
	**SIAs, VW and staffing changes**		
**Sia_vw_month**	Were any SIAs or VW organized in your facility this month?	No one	4
		VW	3
		Measles SIA	2
		other disease SIA	1
**Hcw_vaccinating**	Are HCW still vaccinating at center during SIAs or VW?	always	3
		sometimes	2
		never	1
**Staffing_change**	Do you have a staffing change during SIAs or VW?	increasing number of HCW	3
		no change	2
		decreasing number of HCW	1

Table S9: Geographical characteristics of healthcare facilities visited.				
**Regions**	**Districts**	**Settings**	**Number of centers**	**%**
**Vatovavy Fitovavy**			14	28·6
	Manakara		6	12·2
		Urban	1	2·0
		Rural, main road	2	4·1
		Rural, secondary road	3	6·1
	Vohipeno		8	16·3
		Urban	0	
		Rural, main road	1	2·0
		Rural, secondary road	7	14·3
**Boeny**			18	36·7
	Mahajanga		8	16·3
		Urban	8	16·3
		Rural, main road	0	0·0
		Rural, secondary road	0	0·0
	Marovoay		10	20·4
		Urban	0	
		Rural, main road	5	10·2
		Rural, secondary road	5	10·2
**Toliara**			17	34·7
	Tulear I		9	18·4
		Urban	9	18.4
		Rural, main road	0	0·0
		Rural, secondary road	0	0·0
	Tulear II		8	16·3
		Urban	0	0·0
		Rural, main road	4	8·2
		Rural, secondary road	4	8·2
**Total**			49	100

Table S10: The Lasso regression coefficients. For each timeliness group and for unvaccinated children, factors association with the number of children unvaccinated, vaccinated early (under 9 months) or late (over 12 month).

Variables	Early vaccination	Late vaccination	Unvaccinated children
**(Intercept)**	1·032	-1·190	3·032
**Month**	0·034	0·109	-1·362
**Location**	0·141	0·268	2·749
**Open_center**	0·205	0·216	1·054
**Closing_period**	.	.	.
**Max_travel_time**	-0·241	.	-1·194
**Travel_season**	-0·774	.	.
**Access_storage**	0·036	.	.
**Number_IS**	0·359	0·335	4·770
**Kids_vac_out_IS**	-0·228	-0·172	-0·406
**Outreach_freq**	0·014	.	1·076
**SIA_VW_month**	.	.	.
**HCW_vaccinating**	0·146	.	.
**Staffing_change**	-0·315	-0·062	-2·144

2. Results

1. National data

Table S11: Number of doses delivered by region and year from 2013 to 2016.

Region	N	%	min	max	mean	SD
ALAOTRA MANGORO	208,846	6	0	24,669	870	2,133
AMORON'I MANIA	111,749	3	0	16,622	585	1,257
ANALAMANGA	475,262	13	7	50,193	1238	3,124
ANALANJIROFO	175,167	5	0	30,315	608	1,831
ANDROY	88,192	2	0	7,114	459	783
ANOSY	101,309	3	17	18,854	704	1,567
ATSINANANA	201,297	5	0	23,254	603	1,491
BETSIBOKA	56,549	2	0	15,723	395	1,321
BOENY	109,949	3	2	10,342	382	725
BONGOLAVA	11,6276	3	72	49,149	1199	5,047
DIANA	113,213	3	0	17,392	470	1,146
HAUTE MATSIATRA	185,620	5	0	14,889	780	1,303
IHOROMBE	119,970	3	0	35,802	833	4,220
ITASY	149,137	4	377	51,878	1036	4,279
MELAKY	78,537	2	0	13,327	330	1,331
MENABE	186,305	5	0	27,805	776	3,080
SAVA	147,033	4	25	6,093	766	592
SOFIA	204,137	6	10	21,539	606	1,332
SUD-EST	112,408	3	0	8,210	478	781
SUD-OUEST	189,783	5	0	9,955	438	831
VAKINANKARATRA	334,662	9	0	55,551	996	3,254
VATOVAVY FITOVINANY	237,103	6	0	8,845	823	881
**Total**	3,702,504	100	0	55,551	690	2,112
**Year**						
2013	906,842	24	0	51,878	675	2,076
2014	749,938	20	0	4,863	561	530
2015	663,825	18	0	6,298	497	478
2016	1,380,841	37	0	55,551	1027	3,583
**Total**	3,702,504	100	0	55,551	690	2,112

Table S12: The association between rainy season and number of doses delivered. Using a multivariate linear regression, we explored the relationship between monthly number of doses delivered through routine activities and rainy season adjusted by region, and additional activities (VW or SIA)

	Coefficient	Standard Error	p-value
**Season**			
**Dry**	1	-	-
**Rainy**	-0·076	0·030	0·010*
**Regions**			
**ANALAMANGA**	1	-	-
**ALAOTRA**	-0·380	0·084	<0·001***
**AMORON'I**	-0·747	0·090	<0·001***
**ANALANJIROFO**	-0·971	0·079	<0·001***
**ANDROY**	-1·062	0·100	<0·001***
**ANOSY**	-0·473	0·099	<0·001***
**ATSINANANA**	-0·800	0·076	<0·001***
**BETSIBOKA**	-1·484	0·100	<0·001***
**BOENY**	-1·261	0·079	<0·001***
**BONGOLAVA**	-0·388	0·117	<0·001***
**DIANA**	-0·843	0·090	<0·001***

**HAUTE**	-0·411	0·084	<0·001***
**IHOROMBE**	-1·415	0·100	<0·001***
**ITASY**	-0·150	0·1000	0·131
**MELAKY**	-2·174	0·084	<0·001***
**MENABE**	-1·040	0·084	<0·001***
**SAVA**	-0·167	0·090	0·064
**SOFIA**	-0·722	0·076	<0·001***
**SUD-EST**	-1·623	0·085	<0·001***
**SUD-OUEST**	-1·222	0·071	<0·001***
**VAKINANKARATRA**	-0·114	0·076	0·136
**VATOVAVY**	-0·193	0·080	0·015*
**Additional activity (VW or SIA)**			
**No**	1	-	-
**Yes**	0·926	0·039	<0·001***

Table S13: A comparison of the doses delivered during months before and after a SIA from 2013-2016. Doses were reported as delivered to either 9-12 months years old children or over 12 months years old. Administrative data reported if doses are delivered at the center or outside of the center.

**Total doses delivered**	Months post-SIA	%	Month before SIA	%
**9-12 months**	1,316,654	62·3	1,435,686	94·4
**> 12 months**	797,834	37·7	84,976	5·6
**Total**	2,114,482	100	1,520,335	100
**HF**	395,256	18·7	375,041	24·7
**OHF**	1,719,232	81·3	1,145,621	75·4
**Total**	2,114,482	100	1,520,335	100

Table S14: A comparison of average doses delivered through by month before and after SIA from 2013-2016. Average doses were calculated monthly **from total doses delivered** over the country **during routine immunization months (months with no SIA nor VW)**. Comparison between average number of doses delivered through before a VW were calculated using doses delivered during March, April and September months.

	Months before SIA						Months after SIA					
**Month**	Total doses	Average doses delivered (SD)	9-12 mo (SD)	>12 mo (SD)	HF (SD)	OHF (SD)	Total doses delivered	Average doses delivered (SD)	9-12 months (SD)	+12 months (SD)	HF (SD)	OHF (SD)
**Jan**	94231	432.2 (418.1)	421.8 (408.1)	10.4 (100.1)	359.1 (352.7)	72.0 (147.4)	80858	369.2 (364.4)	366.4 (363.6)	2.8 (9.7)	331.8 (334.6)	36.6 (71.8)
**Feb**	95022	441.9 (388.3)	438.7 (386.9)	3.3 (11.3)	357.7 (325.2)	83.4 (149.5)	90147	411.6 (524.4)	406.4 (514.9)	5.3 (36.3)	364.8 (480.7)	46.4 (106.3)
**March**	96820	450.3 (371.5)	447.31 (370.6)	3.0 (8.7)	378.0 (326.3)	71.3 (103.8)	99907	460.4 (440.3)	451.8 (418.6)	8.6 (38.9)	377.3 (349.7)	81.2 (137.2)
**April**	55117	501.1(362.8)	496.7 (360.3)	4.4 (10.8)	404.9 (327.7)	95.5 (99.2)	111054	507.1 (493.0)	489.6 (426.8)	18.9 (134.7)	414.8 (384.4)	92.3 (181.3)

**May**	40961	372.4 (336.0)	366.3 (332.0)	5.9 (17.5)	315.3 (303.6)	56.3 (108.8)	NA	NA	NA	NA	NA	NA
**June**	91020	415.6 (376.2)	410.5 (369.1)	5.2 (16.1)	333.1 (317.9)	81.6 (119.3)	91510	421.7 (371.4)	410.4 (367.1)	11.3 (35.3)	332.2 (313.2)	88.3 (128.6)
**July**	101527	463.6 (379.1)	457.4 (374.1)	6.1 (14.7)	367.7 (322.6)	95.6 (126.5)	94756	436.7 (391.9)	429.3 (391.4)	7.4 (29.6)	343.4 (327.8)	90.6 (138.2)
**Aug**	103438	470.2(346.4)	463.7(345.4)	6.4 (21.5)	354.1 (304.8)	116.7 (130.5)	101027	463.4 (531.9)	459.1 (531.5)	4.4 (12.8)	361.9 (474.7)	99.5 (147.2)
**Sept**	103529	474.9 (387.2)	470.7 (382.1)	4.2 (12.3)	349.7 (302.1)	124.0 (144.2)	106039	502.6 (399.9)	494.8 (397.5)	7.7 (20.6)	368.0 (334.1)	129.6 (156.1)
**Nov**	44699	413.9(361.4)	406.6 (358.8)	7.3 (26.9)	308.3 (309.3)	104.8 (140.3)	195895	599.1 (545.6)	555.9 (459.3)	43.1 (234.3)	446.7 (433.1)	153.5 (188.2)
**Dec**	41915	391.7 (355.9)	385.7 (353.2)	6.0 (33.7)	287.2 (301.3)	103.2 (152.6)	146423	451.9 (361.3)	434.4 (343.6)	17.5 (133.4)	339.5 (296.7)	110.4 (150.1)

National data provide information about place and age for doses delivered. We retrieved from overall doses the one delivered during additional activities months and considered that doses delivered during non-additional activities months were delivered through routine immunization. Average number were calculated by diving total number of doses delivered by the number of months they were delivered. Similarly, monthly average number of doses delivered through routine immunization were calculated as total doses delivered per month/number of months occurrences. NA indicates that no data were considered as delivered through routine immunization as a VW occurred during the month.

2. Individual data results

B

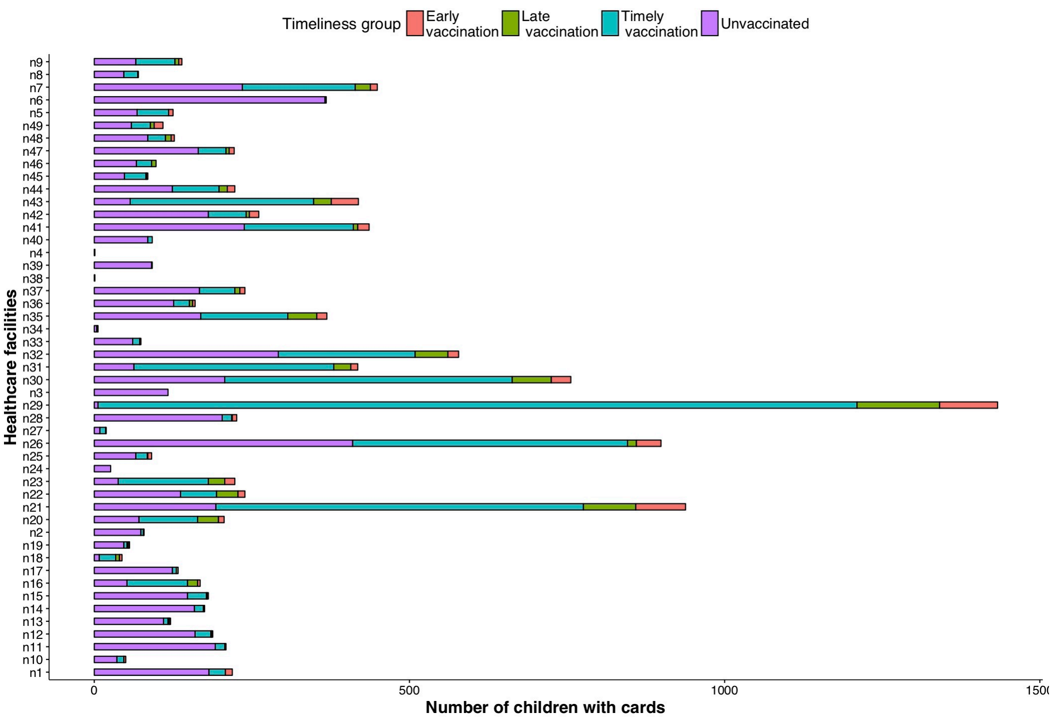

Figure S6: The individual vaccination card data repartition. For each health facility, we identified the number of early, late, timely and unvaccinated children.

Welch-corrected ANOVA results for each timeliness group are presented in Table S15. Since only early vaccination variation was significant, we performed a Games-Howell post-hoc test only for this timeliness group.

Table S15: ANOVA results exploring the relation between percentage of children with cards vaccinated early through RI and period post activity

		Df	Sum square	Mean square	F value	p-value
**Early**	Period post activity	2	6·329e-03	3·165e-05	3·73	0·004*
	Residuals	16·8	0·001	0·389e-05		
**Timely**	Period post activity	2	0·004	0·002	0·445	0·64
	Residuals	15·1	0·141	0·005		
**Late**	Period post activity	2	3·231e-05	1·615e-04	3·505	0·06
	Residuals	10·9	16·577e-05	6·376e-05		

TukeySD test for early vaccination

	Mean difference	Confidence Intervalle		p
		Lower bound	Upper bound	
post-May VW/ post-SIA	-0·012	-0·020	-0·004	0·002
post-October VW/ post-SIA	-0·010	-0·018	-0·003	0·007
post-October VW/post-May	0·002	-0·005	0·008	0·767

We explored the difference between urban and rural settings as shown in Fig S6. The average age of vaccination for children from rural areas was higher than in urban settings (10.1 vs 9.8 months old). In each timeliness group,

the variations observed at the global level were higher in rural areas. Of children with cards who received timely vaccination, the proportion was significantly lower

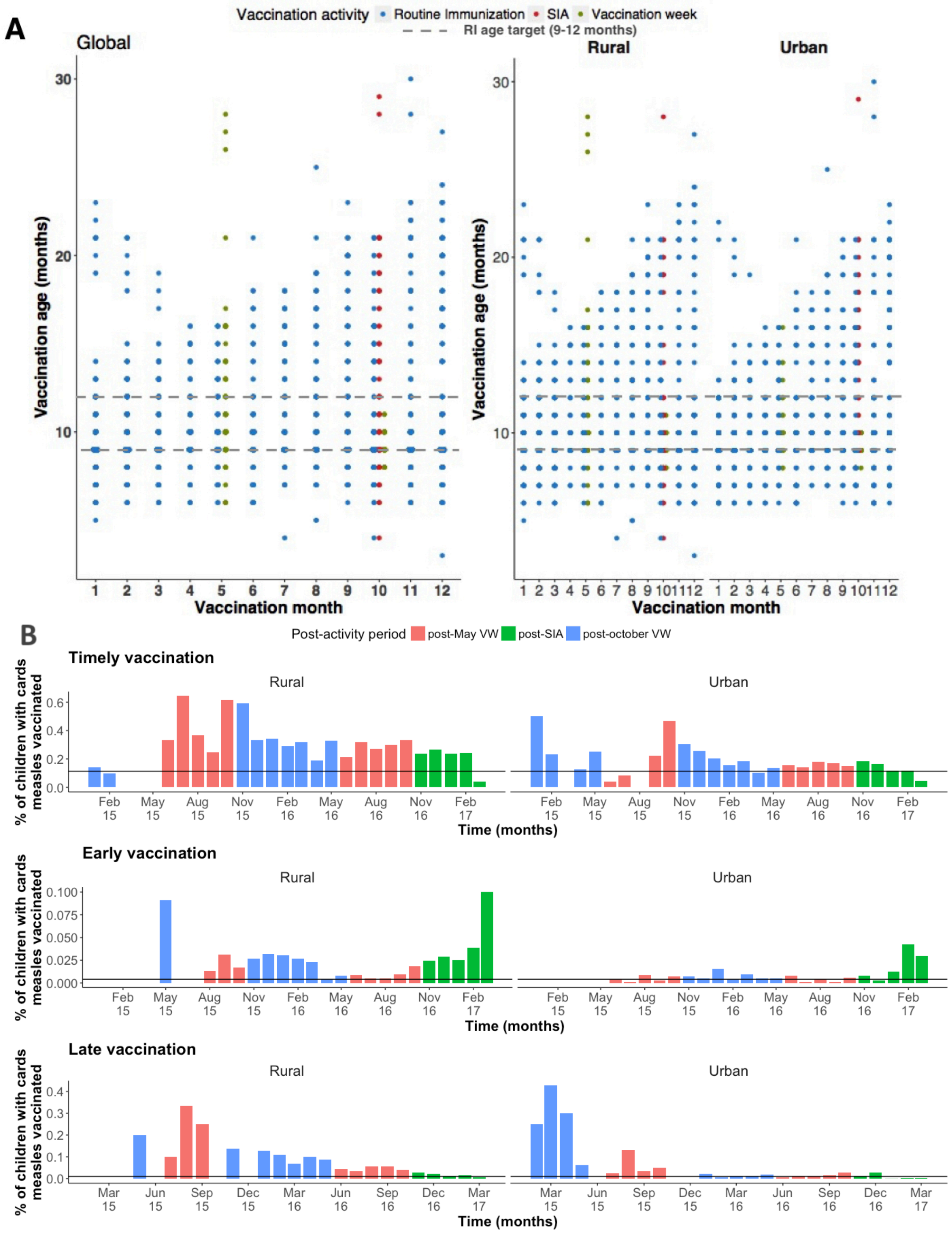

Figure S7 – The spatial heterogeneity in children vaccinated. A) The age distribution in urban and rural setting. B) The percentage of vaccinated children with cards according to their timeliness group

3. Health care factors associated to missed vaccination

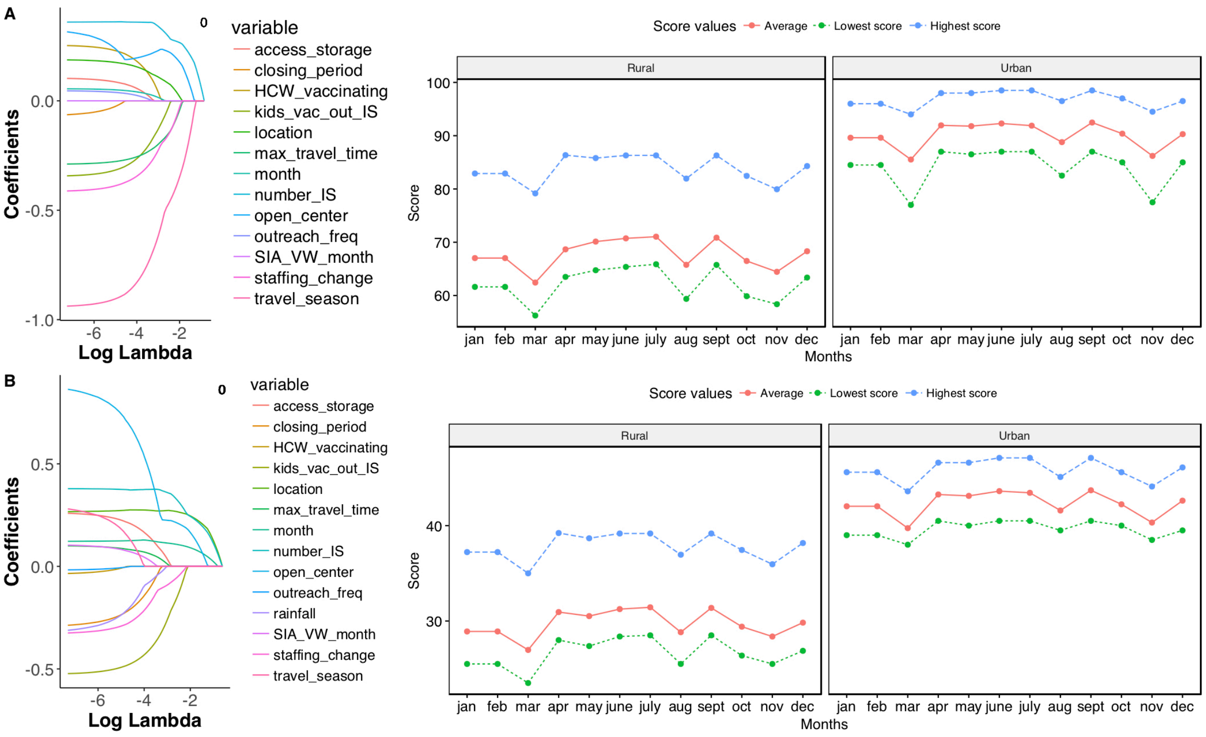

Figure S8– The Lasso results and monthly score for early and late vaccinations. ∫Both early and late vaccination are associated with the number of immunization sessions and the ability to maintain center open through the year. Early vaccination is associated with ability to keep vaccinating at center while ongoing additional activities whereas late vaccination is associated with the frequency of at-door vaccination. As for unvaccinated children, scores are lower in rural areas than in urban settings. However, the same pattern with a decreasing access in March, August, October and November is found.

Bibliography

1. Macron C, Richard Y, Garot T, Bessafi M, Pohl B, Ratiarison A, et al. Intraseasonal Rainfall Variability over Madagascar. Mon Weather Rev. 2015 Jul 29;144(5):1877–85.

2. WHO. The immunization data quality sel-assessement (DQS) tool. 2005.

3. Odgers DJ, Tellis N, Hall H, Dumontier M. Using LASSO Regression to Predict Rheumatoid Arthritis Treatment Efficacy. AMIA Summits Transl Sci Proc. 2016 Jul 20;2016:176–83.
